# A Microfluidic Probe Integrated Device for Spatiotemporal 3D Chemical Stimulation in Cells

**DOI:** 10.3390/mi11070691

**Published:** 2020-07-16

**Authors:** Kenta Shinha, Wataru Nihei, Hiroshi Kimura

**Affiliations:** 1 Department of Mechanical Engineering, School of Engineering, Tokai University, 4-1-1 Kitakaname, Hiratsuka, Kanagawa 259-1292, Japan; shinba04@gmail.com (K.S.); nihei.wataru@tokai.ac.jp (W.N.); 2 Micro/Nano Technology Center (MNTC), Tokai University, 4-1-1 Kitakaname, Hiratsuka, Kanagawa 259-1292, Japan

**Keywords:** microfluidic probe, microenvironment, microfluidic device, cell culture

## Abstract

Numerous in vitro studies have been conducted in conventional static cell culture systems. However, most of the results represent an average response from a population of cells regardless of their local microenvironment. A microfluidic probe is a non-contact technology that has been widely used to perform local chemical stimulation within a restricted space, providing elaborated modulation and analysis of cellular responses within the microenvironment. Although microfluidic probes developed earlier have various potential applications, the two-dimensional structure can compromise their functionality and flexibility for practical use. In this study, we developed a three-dimensional microfluidic probe integrated device equipped with vertically oriented microchannels to overcome crucial challenges and tested the potential utility of the device in biological research. We demonstrated that the device tightly regulated spatial diffusion of a fluorescent molecule, and the flow profile predicted by simulation replicated the experimental results. Additionally, the device modulated the physiological Ca^2+^ response of cells within the restricted area by altering the local and temporal concentrations of biomolecules such as ATP. The novel device developed in this study may provide various applications for biological studies and contribute to further understanding of molecular mechanisms underlying cellular physiology.

## 1. Introduction

Cells communicate with one another by transmitting signals to maintain physiological homeostasis [1,2]. The intercellular signal transmission is initiated by the binding of signaling molecules to receptors and the subsequent influx of ions through various channels in the plasma membrane, inducing multiple cellular responses. For example, calcium ions (Ca^2+^) play an important role in intercellular communications associated with induction of differentiation and cardiac contraction. To date, several methods have been developed and employed to study the local cell-cell interactions [3,4,5,6,7,8,9,10]; however, it remains challenging to investigate the intercellular communication due to difficulties in spatiotemporal control of the microenvironment surrounding the cells of interest. Therefore, it is important to develop methods and tools to dissect the cell-cell interactions by controlling the local microenvironment.

To control the local microenvironment, a concentration gradient of injected molecules was created by gradient generators in combination with laminar flow within microchannels [11,12,13,14], and the idea was applied for high-throughput cell-based assays for biomedical research [15]. Although the methods using laminar flow have great spatial resolution in a vertical flow direction, the spatial resolution in the horizontal flow direction is quite low. A microfluidic probe (MFP) is a non-contact technology that has been proposed to regulate the microenvironment surrounding the targeted cells [16,17,18]. An MFP has two microchannels, one for injection and the other for aspiration. Diffusion of injected reagents can be regulated by maintaining a balance between injection and aspiration flow rates. Juncker et al. used a MFP to perform protein patterning on a substrate, localized chemical stimulation, and for selective staining of the cells [19,20,21,22,23,24,25,26,27]. Although MFPs are simple devices used in open-culture systems like conventional cell culture dishes, the integration of MFP technology into microfluidic devices could be highly beneficial for cell-based assays.

We previously developed a microfluidic device integrated with an MFP [28]. The device has flow channels on the wall of a cell culture chamber to replicate the function of an MFP, providing live-cell imaging with localized chemical stimulation. Moreover, it maintains an in vivo like environment using applied mechanical stimuli such as shear stress [29,30]. However, the horizontal flow direction limited the function of the device. Furthermore, the flow was directly formed after starting perfusion on the cells. Thus, only simple experiments such as cell staining and cell harvest can be performed using the device. This study aimed to develop a three-dimensional MFP integrated device to overcome the limitations mentioned above for investigating cellular physiology. We developed a microfluidic cell culture device with an MFP attached to the roof of a cell culture chamber that can provide arbitrary stimulations on the X and Y axes. We demonstrated that the device can spatiotemporally regulate chemical stimulation and the subsequent physiological cellular response in the microenvironment. These results suggested that the device can potentially be applied for biological research.

## 2. Materials and Methods 

### 2.1. MFP Integrated Device

**Figure 1:**
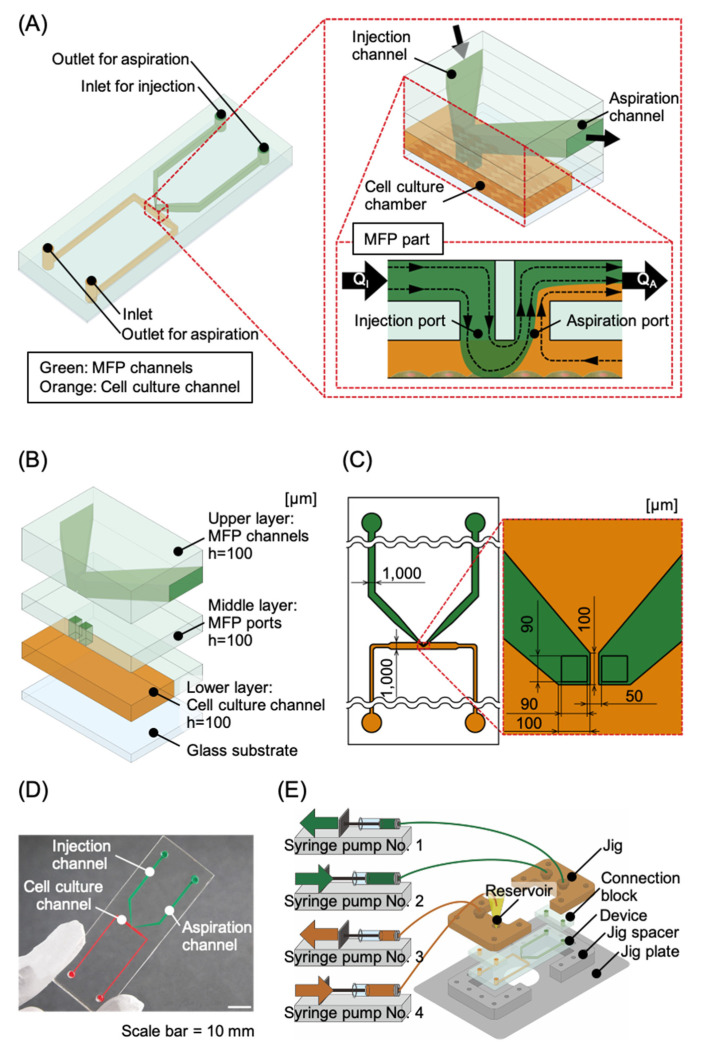
Conceptual diagram of the microfluidic probe (MFP) integrated device. (**A**) MFP integrated device design. The device consists of an MFP channel (indicated in green) and a cell culture channel (indicated in orange). To obtain a localized chemical stimulation area, flow ratios are set such that Q_I_ < Q_A_. (**B**) The device is composed of four layers: upper, middle, lower, and a glass substrate. The upper, middle, and lower layers correspond to the MFP channels, the MFP ports, and the cell culture channel, respectively. (**C**) Scale diagram of the channel section of the device. The major portion of each MFP channel and the cell culture chamber have a channel width and height of 1 mm and 100 μm, respectively. (**D**) Photograph of the device made from polydimethylsiloxane (PDMS) using photolithography. An inlet and outlet are fabricated at each microchannel in this device. (**E**) Experimental setup. Thick PDMS connection blocks are attached to the locations of the inlets and the outlets for installation in a jig connected to syringe pumps. Syringe pumps no. 1 and no. 2 are connected to the MFP channels and are used for fluid control in the chemical stimulation area. Syringe pumps no. 3 and no. 4 are connected to the cell culture channel, and are used for cell culture, solution replacement, and other related activities. The device with the jig is installed onto the stage of a confocal microscope.

A novel MFP integrated device was developed in this study. It comprises a pair of injection and aspiration ports with each channel (green colored channels in Figure 1A) installed onto the roof of a cell culture chamber, which ensures localized chemical stimulation. The cell culture chamber is within a cell culture microchannel, which is connected to an inlet with a reservoir and an outlet for cell seeding and medium supply (orange colored channel in Figure 1A). The localized chemical stimulation area formed using an MFP depends on the ratio of injection to aspiration flow, distance between the injection and suction ports, size of the ports, and distance between the cells and the ports [16,21,31,32,33]. The microchannels were designed based on these aspects (Figure 1B,C). The device was installed into a jig with interfaces such as tube connecters and a reservoir for high precision fluidic control.

### 2.2. Fabrication

The device consisted of four layers: an upper layer, a middle layer, a lower layer, and a glass substrate comprising the MFP channels, MFP ports, the cell culture channel, and the bottom, respectively. The upper, middle, and lower layers were composed of polydimethylsiloxane (PDMS), and fabricated using conventional soft lithography [34,35,36]. Master molds for the three PDMS layers were manufactured using photolithography. Four-inch chrome (Cr) mask substrates were designed using CAD software (AutoCAD 2016, Autodesk, Inc., San Rafael, CA, USA) (Figure S1A–C) and fabricated using a micro-pattern generator (μPG101, Heidelberg Instruments Inc., Heidelberg, Germany). A negative photoresist (SU-8 2100, MicroChem, Westborough, MA, USA) of 1.9 g (100 μm of the upper and lower layers) and 2.5 g (160 μm of the middle layer) was spin coated onto the Cr mask substrates directly to fabricate high-resolution microstructures. The photoresist layers on the substrates were baked at 95 °C for 30 min (the upper and lower layers) and 20 min (the middle layer) using a hot plate as the soft bake, and then baked again at 95 °C for 5 min (the upper and lower layers) and 12 min (the middle layer) as the post exposure bake to form microstructures on the substrates by exposing them to an ultraviolet lamp of 240 mJ/cm^2^ (the upper and lower layers) and 260 mJ/cm^2^ (the middle layer). The substrates were dipped in the MicroChem SU-8 developer for 15 min with shaking to develop the exposed layers, and then washed twice with isopropanol and ion-exchanged water. The surface of the substrates was coated with CHF_3_ as a mold lubricant using reactive ion etching (RIE-10NR, Samco Inc., Kyoto, Japan). 

The substrates were used as master molds for PDMS (SILPOT 184, Dow Corning Toray, Tokyo, Japan). A 10:1 mixture of PDMS and a polymerization agent was poured onto the master molds in the upper layer, and heat cured in an oven at 75 °C for 2 h. The mixture was spin coated at 600 rpm for 20 s with air blowing onto the master molds in the middle and lower layers to make through-structures and heat cured in an oven at 75 °C for 2 h. The cured PDMS was peeled off from the master mold to produce chips to which the microstructures were transferred. Microchannel inlets and outlets (2 mm diameter each) were fabricated on the upper layer of the PDMS chips using a trepan (BP-L20K, Kai). The surfaces of the three layers of the PDMS chips and a cover glass plate were activated using a plasma cleaner (PDC-32G, Harrick Plasma, Ithaca, NY, USA) and then aligned using pure water and permanently bonded together to assemble the MFP integrated device [37]. The fabricated device is shown in Figure 1D. Furthermore, PDMS connection blocks were attached at the locations of the ports using plasma bonding to set the device into the jig (Figure 1E). The jig was made of poly ether ether ketone (PEEK) and fabricated with machine processing (Figure S2A). The jig spacer and plate were made of duralumin and fabricated with machine processing (Figure S2B,C). The jig, jig plate, and jig spacer were fixed with screws.

### 2.3. Microfluidic Control Setup and Method

The MFP integrated device was set in the jig. To control the MFP functionality, the inlets and the outlets of the MFP and cell culture channels were connected to four independent high-resolution syringe pumps (Microfluidic System Works) using fluorinated ethylene propylene (FEP) tubes (OD: 1/16 inch and ID: 1/100 inch, Upchurch Scientific, Oak Harbor, WA, USA). The reservoir for storing the medium was installed at the inlet of the cell culture channel. The flow rate in the MFP channels was controlled using the syringe pumps no. 1 and no. 2, which were connected to its inlet and outlet, respectively, as shown in Figure 1E. Using these syringe pumps, the solution containing a humoral factor was injected at flow rate Q_I_ into the injection port of the MFP channel and was aspirated at flow rate Q_A_ from the suction port of the MFP channel, as shown in Figure 1A. The diffusion of the humoral factor in the solution could be suppressed by maintaining Q_I_ < Q_A_, resulting in a high-resolution localized exposure area for the humoral factor within the cell culture chamber. Simultaneously, the amount of medium corresponding to the difference between Q_A_ and Q_I_ was discharged from the cell culture channel, allowing for a sufficient amount of the medium to be stocked in the reservoir. The syringe pump no. 3 was used for aspirating the solution from the reservoir required for cell inoculation and medium replacement in the cell culture channel. The syringe pump no. 4 was used for supplying the medium into the reservoir. The device with the jig was installed into a stage top incubator maintained at 37 °C in a humidified atmosphere containing 5% CO_2_ on the stage of a confocal microscope (A1, Nikon, Tokyo, Japan) in Tokai University Imaging Center for Advanced Research (TICAR). 

### 2.4. Verification of MFP Function

Functions of the MFP within the device were verified by comparing simulation and experiment. Computational fluid dynamics (CFD) analysis was performed using the general-purpose physics simulation software ANSYS Fluent 17.1, which is based on the finite element method (FEM), to estimate the chemical stimulation area using parameters such as the channel design and the flow ratio. We used the pressure-based solver in ANSYS Fluent. The multiphase model and the viscose model were set to “Mixture” and “Laminar”, respectively, to evaluate the flow and the diffusion of substrates in the cell culture chamber. The density, viscosity, and other parameters of the solution at 37 °C were set to the preset values of the water in ANSYS Fluent 17.1 (Ansys, Inc., Canonsburg, PA, USA). The SIMPLE method was set as the solution methods, the “Max Iterations/Time Step” was set to 25, and the mesh topology was tetrahedron (Table S1).

The microchannel model geometry was built similar to the device that we manufactured. In the simulation, the flow ratios were set to the same values as those in the experiment. Fluorescein sodium salt was used as the humoral factor, with a diffusion coefficient of D = 4.9 × 10^−10^ m^2^/s [38] and a concentration of 100 μM. The chemical stimulation area on the bottom of the cell culture chamber was measured from the images obtained from the simulation results, using image-processing software (ImageJ, NIH, Bethesda, MD, USA) in steady-state under each condition with a threshold concentration which was 10% of the concentration of the original solution. 

An experiment was performed using the fabricated device to verify the validity of the simulation results. The device was installed on the stage of a microscope, and the ports were connected to the four syringe pumps, as described previously. Fluorescein sodium salt (Uranin, F0096, Tokyo Chemical Industry, Tokyo, Japan) (100 μM) was used as the humoral factor, and fluorescence images were taken from the bottom of the cell culture chamber around the MFP ports using the confocal microscope. The flow ratios (Q_I_:Q_A_) were set to 0.10:1.00–0.33:1.00 µL/min. The chemical stimulation area in the channel was measured quantitatively from the fluorescence images using ImageJ with a threshold concentration of 10 μM. Fluorescence intensities of the images obtained from the experiment were calculated according to a standard curve, which we obtained prior to the experiment using different concentrations of fluorescein injected into the device; the linearity of the curve was maintained from 10 to 70 μM. Thus, a 10 μM threshold was set for binarization for area calculation. 

### 2.5. Cell Culture

Madin-Darby canine kidney (MDCK) cells were obtained from the JCRB Cell Bank. The cells were cultured and maintained at 37 °C in an incubator in a humidified atmosphere containing 5% CO_2_. The culture were grown in Dulbecco’s modified Eagle medium (12320-032, Gibco) supplemented with 10% fetal bovine serum (FBS, Biowest, Nuaillé, France), 1% non-essential amino acid solution (11140–050, Thermo Fisher, Waltham, MA, USA), and 1% antibiotic and antimycotic solution (161–23181, Fujifilm Wako, Osaka, Japan). Chinese hamster ovary (CHO-K1) cells (JCRB Cell Bank, Osaka, Japan) were maintained at 37 °C in an incubator in a humidified atmosphere containing 5% CO_2_. The cells were maintained in Ham’s F-12 medium (087–08335, Wako Pure Chemical Industries, Osaka, Japan), supplemented with 10% fetal bovine serum (FBS, Bio West, Japan), 1% non-essential amino acid solution (11140–050, Thermo Fisher, USA), and 1% antibiotic and antimycotic solution (161–23181, Fujifilm Wako, Japan).

The devices were sterilized by filling them with 70% ethanol for 60 min and then rinsing with Milli-Q water before being used. The cell culture channel was coated with collagen by filling the channel with 0.1 mg/mL collagen type I-P (634-00663, Nitta Gelatin Inc., Osaka, Japan) solution for 2 h, followed by a rinse with PBS. The cells were seeded at a density of 1.0 × 10^5^ cells/cm^2^ by introducing the cell suspension to the reservoir by aspirating at the rate of 5.00 µL/min using syringe pump no. 3 (Figure 1D). Next, the device was placed in the incubator for 2 h to allow the cells to adhere to the bottom surface of the cell culture channel. Following cell adhesion, the culture medium stored in the reservoir was aspirated at a flow rate of 0.1 μL/min using syringe pump no. 3 for a perfusion culture. Syringe pump no. 4 was used for supplying the culture medium into the reservoir at a flow rate of 0.1 μL/min.

### 2.6. Evaluation of Cell Response by Local Chemical Stimulation

We performed experiments of chemical stimulation on cells to evaluate the potential utility of the device for the assessment of physiological response induced by a signaling molecule. In this experiment, we observed the changes in Ca^2+^ concentration in MDCK cells due to spatiotemporal chemical stimulation. Adenosine triphosphate (ATP; R0441, Thermo Fisher, USA) was used at a concentration of 75 μM as the humoral factor, which induces an increase in Ca^2+^ concentration. The flow ratios (Q_I_:Q_A_) were set to 0.10:1.00, 1.00:10.00, and 2.00:10.00 µL/min. The change in Ca^2+^ concentration in the cells was measured using a fluorescent Ca^2+^ indicator, Fluo-4 AM (Calcium Kit-Fluo-4, Dojindo, Kumamoto, Japan). After the introduction of Fluo-4 AM into the cells, the chemical stimulation was initiated in each flow condition. The fluorescence intensity of Fluo-4 AM in the cells was observed using a confocal microscope and quantitatively measured from the images using ImageJ. The region of interest (ROI) corresponding to the chemical stimulation area was estimated by simulation. In the simulation, the flow ratios were set to the same values as mentioned in this experiment. A diffusion coefficient of ATP was set to D = 3.0 × 10^−10^ m^2^/s [3].

### 2.7. Cell Collection

It is important to evaluate chemically stimulated cells using qPCR and other methods. For this, the cells should be collected spatially from the cell culture chamber. In this experiment, we investigated whether the cells can be collected site-selectively from the chamber. CHO-K1 cells were seeded onto the chamber according to the procedure described above and were cultured to be confluent. A 0.25% *w*/*v* trypsin/1 mM EDTA·4Na solution with phenol red (T4049, Sigma, St. Louis, MO, USA) was injected at flow ratios (Q_I_) of 0.10–0.33 μL/min from the injection port of the MFP channel, and it was simultaneously aspirated at a flow rate (Q_A_) of 1.0 μL/min. In this manner, the cells were exposed to a site-localized trypsin solution and collected after desquamation. After the collection experiment, the cells were cultured for 2 h to evaluate the effect of trypsin on the surrounding cells.

## 3. Results and Discussion

### 3.1. Flow Control Experiment and Simulation

**Figure 2:**
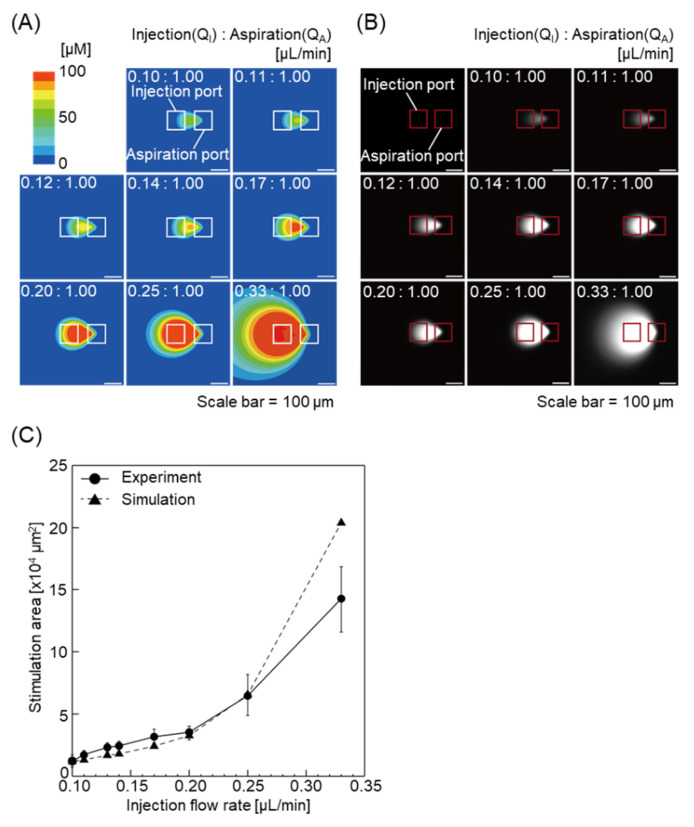
Comparison of experimental results with that of the simulation model to evaluate functionality of the device. (**A**) Fluid analysis simulation results using ANSYS Fluent for evaluation of the MFP functionality. Simulation results for the chemical stimulation area when the flow ratios (Q_I_:Q_A_) are 0.10:1.00 to 0.33:1.00 μL/min. The chemical stimulation area increases with the flow ratio. (**B**) Fluorescence images of the chemical stimulation area and its vicinity when the flow ratios are the same as the simulation. (**C**) Graph comparing the simulation results and the experimental results performed using the device. The results were almost identical. Means are values between maximum values and minimum values.

To evaluate the function of the MFP, we first performed a FEM-based simulation to investigate the chemical stimulation area of fluorescein, which served as a model for the fluorescence molecule. The flow ratio (Q_I_:Q_A_) was set at 0.1:1.00 to 0.33:1.00 μL/min. As the injection flow rate, Q_I_, in the MFP channel increased, the stimulation area of fluorescein expanded (Figure 2A). The numerical simulation results also indicated that concentration gradients were formed depending on the flow ratio in the confined zone (Figure 2A). We then performed flow control experiments using the MFP. Consistent with the numerical simulation results, the exposed area was found to be regulated by the flow ratio (Figure 2B). There was a chemical concentration gradient in the chemical stimulation area. Although the concentration gradient decreased due to an increase in the flow rate, the shear stress also increased, which might affect cell functions. The chemical concentration gradient and the shear stress are related to the transactions. It is difficult to expose cells within the chemical stimulation area to chemical factors without any shear stress using the MFP. The chemical concentration gradient in the chemical stimulation area and the shear stress can be estimated by the numerical simulation. Thus, the MFP should be used with optimal flow rates depending on the cell species and phenomena. Furthermore, the chemical stimulation area was quantified in both experiments. As shown in Figure 2C, the flow rate-dependent stimulation area was almost equivalent in the case of both the simulation and the experiment from 0.1:1.00 to 0.25:1.00 μL/min except 0.33:1.00 μL/min. 

We simulated the chemical stimulation area using three different mesh sizes at the flow ratio (Q_I_:Q_A_) 0.33:1.00 to evaluate the effect of the mesh size. “Proximity Min Size”, “Max Face Size”, and “Max Tet Size” were set to 100 times and 1000 times the original size that we used in this study. The results showed that the larger the mesh sizes, the larger were the estimated chemical stimulation areas, and the effect of the mesh size was significant, especially in a low concentration of a chemical stimulation factor (Figure S3). It is well known that the smaller the mesh size gets, the higher the accuracy we have in the FEM. The mesh size and density were small and high around the MFP ports and were gradually larger and lower, respectively, depending on the distance from the MFP ports in ANSYS Fluent. This aspect caused a significant error when the chemical stimulation area was larger than the area in the flow ratios 0.25:1.00, especially in a low chemical concentration area. Although we could obtain more accurate results using the smaller mesh size, the mesh size used in this study was the smallest that we could perform using the workstation (CPU: Intel Xeon CPU E3-1271 v3 @3.6 GHz, RAM: 32.0 GB). Thus, we conclude that the estimated chemical stimulation area in the flow ratios (Q_I_:Q_A_) less than 0.25:1.00 are reliable in the simulation condition.

The diffusion coefficient directly affects the diffusion range of molecules. The diffusion range increases in proportion to the diffusion coefficient in the same flow ratio. Fluorescein, serving as a model for the fluorescence molecule in the present study, has a low molecular weight and is widely used as a highly diffusible molecule. Moreover, the stimulation area of the fluorescein was almost equivalent in both simulation and experiment. Thus, the device can be applied to highly diffusive chemicals. 

### 3.2. Evaluation of Cell Response via Local Chemical Stimulation

**Figure 3:**
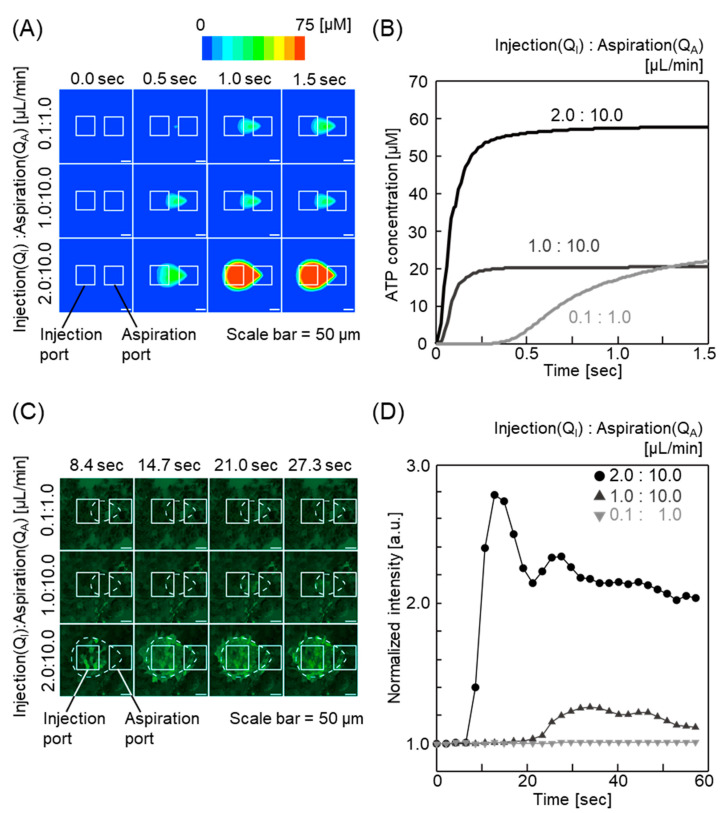
Results of chemical stimulation on Madin-Darby canine kidney (MDCK) cells using the device. (**A**) Fluid analysis simulation results using ANSYS Fluent to estimate the stimulation area of adenosine triphosphate (ATP). Time-dependent changes in the stimulation area are shown in each flow ratio; 0.1:10.0, 1.0:10.0, and 2.0:10.0. (**B**) Time-dependent changes in mean ATP concentration in the stimulation area calculated using ANSYS Fluent. (**C**) Time-lapse fluorescence images of spatial chemical stimulation of the MDCK cells in the cell culture chamber using ATP solution. The flow ratios are the same as in the simulation. Areas surrounded by broken lines are the stimulation area estimated by the simulation. Green fluorescence shows increased Ca^2+^ concentration using Fluo-4 AM. (**D**) Fluorescence intensity of Fluo-4 caused by Ca^2+^ concentration increase in the MDCK cells.

Next, experiments were performed to evaluate the potential utility of the MFP for assessment of physiological response in cells induced by signaling molecules. We examined ATP as the signaling molecule in FEM-based simulation and evaluated ATP-dependent Ca^2+^ response in the MFP. The simulation results showed an increase in stimulation area of ATP with increasing flow rate (Figure 3A). Furthermore, theoretical ATP concentrations over time were calculated for each flow rate (Figure 3B). The ATP gradients within a ROI were converted to grayscale images; 256 tone levels were assigned from a minimum (0 μM) to maximum value (75 μM) by the simulation software. The intensity values of the stimulated area were averaged and normalized to the value at 0 s using the NIH ImageJ software. The average tone levels were converted to ATP concentration by multiplying by 0.29297. At a flow rate of 2.0:10.0 μL/min and 1.0:10.0 μL/min, the ATP concentration increased almost linearly and reached a plateau within 1 s (58 and 21 μM, respectively), and for a flow rate of 0.1:1.0, the ATP concentration gradually increased (Figure 3B). These results suggest that control of the flow rate and flow ratio in the MFP enables spatiotemporal regulation of the local ATP concentration within the microenvironment. We further investigated the physiological response induced by ATP in a cell model. ATP induces an increase in intracellular Ca^2+^ in MDCK cells [39]. We measured the ATP-induced Ca^2+^ response using Fluo-4 AM, a calcium indicator that displays an increase in fluorescence intensity on Ca^2+^ binding [40]. MDCK cells treated with ATP showed a flow rate-dependent increase in intercellular Ca^2+^ (Figure 3C). The relative fluorescence intensity was quantified for each flow rate. The average fluorescence intensity values in the ROI were calculated and normalized to the value at 0 s using the NIH ImageJ software (Figure 3D). Consistent with the predicted ATP concentrations in Figure 3B, MDCK cells showed rapid elevation in fluorescence intensity at a flow rate of 2.0:10.0 μL/min (Figure 3D). Nevertheless, the estimated maximum ATP concentrations for 1.0:10.0 μL/min and 0.1:1.0 μL/min were similar in that the cells showed partial or no response to ATP stimulation (Figure 3D). A key difference between the two flow conditions was the flow velocity due to different flow rates. Previous studies indicate that shear stress induces Ca^2+^ response in MDCK cells [41,42,43]. These observations, taken together, suggest that the different Ca^2+^ response was caused by the increased shear stress associated with the increased flow rate. Interestingly, we observed Ca2^+^ response caused by both ATP concentrations and microfluidic shear stress using the device. Together, the strength and duration of the signaling response can be modulated by controlling the flow rate and flow ratio in the MFP. In this study, we showed that it is difficult to observe quick cell responses with conventional methods, while our device is advantageous in this respect. A key advantage of our device is that it can observe both the effects of concentration and shear stress, unlike conventional static cell culture systems. We tested that the stimulation area could be kept stable over 24 h using this device. Thus, the device can also be applied for chronic stimulation. Ca^2+^ signaling is involved in fertilization, embryogenesis, organogenesis, and other developmental events in a highly coordinated manner [44,45,46,47]. These processes require three-dimensional control of the local environment such as gradients of biomolecules, flow conditions, and cell-cell communications. Moreover, the MFP can collect local stimulated cells by dissociating adherent cells for further analysis (Supplementary Figure S4). Figure S4 shows that the cells within the cell culture chamber were collected locally by controlling the chemical stimulation area in each flow ratio. Cells present in the chemical stimulation area before exposure lost their adhesion ability after being exposed to trypsin. A total of 1.4 × 10^4^ to 9.0 × 10^4^ µm^2^ cells were collected when the flow ratios (Q_I_:Q_A_) were set to 0.10:1.00 and 0.25:1.00 µL/min respectively, and 70 to 450 cells could be harvested (assuming a cell size of 200 μm). The areas where the cells got detached corresponded to the chemical stimulation areas controlled by the flow ratios. Moreover, the cells surrounding the detached cells were not affected by the trypsin. The detached cell could be collected through the suction port. Therefore, we concluded that the cells can be collected site-selectively from the chamber, and evaluated via qPCR and other methods. Recent advances in single-cell analysis have contributed significantly to a better understanding of cellular physiology and pathogenesis of diseases [48,49,50,51]. Sarker et al. lysed single adherent cells and subsequently analyzed enzymatic activity in an MFP at the single-cell level. Thus, an MFP including the three-dimensional MFP developed in this study can also be applicable for numerous biological studies that require three-dimensional regulation of the microenvironment [26].

It is well known that many substances adsorb and adhere to the PDMS surface. Whether a substance is adsorbed on PDMS depends on the characteristics of the substance. For example, rhodamine B is a substance that is easily attached, while uranin (used in this study) is a substance that is relatively difficult to adsorb. Thus, using PDMS is not a problem for the functionality of this device. Rather, PDMS can be used to realize complex shapes at the laboratory level and has sufficient functions to allow the study of cellular mechanism and function. Blocking with FBS is also effective to some extent to avoid adsorption. Several coating techniques have also been proposed to prevent adsorption and attachment [52,53]. In the future, the usefulness of this device may be further enhanced by coating PDMS devices or fabricating devices of glass or polystyrene. These data demonstrated that the three-dimensional MFP integrated device spatiotemporally regulates physiological responses by controlling the extracellular microenvironment, providing an advanced method and tool for analyzing local cellular signaling response. 

## 4. Conclusions

This study reports the design, fabrication, and practical applications of the three-dimensional MFP integrated device in studying cellular physiology. We predicted the flow profiles of a fluorescent molecule in the fabricated device via FEM-based simulation, and experimental data were in accordance with the simulation results. Control of flow rate and flow ratio in the microchannels regulated spatial diffusion of an injected fluorescent molecule within a restricted area. Furthermore, we verified that the device not only modulated spatial diffusion of biomolecules but also the temporal concentration within a region of interest, resulting in the tightly regulated physiological response of cells in a microenvironment.

## Supplementary Materials

The following are available online at https://www.mdpi.com/2072-666X/11/7/691/s1, Figure S1: The dimensions of the MFP integrated device designed with CAD software, Figure S2: The dimensions of the jig designed with CAD software, Figure S3: Evaluation of the effect of mesh size on simulation results, Figure S4: Bright-field images of CHO cells when exposed to trypsin using the MFP on the device to harvest cells, Table S1: Detailed conditions for mesh size.
