# Supplementary material for: A Microfluidic Probe Integrated Device for Spatiotemporal 3D Chemical Stimulation in Cells
Source: Micromachines (Basel). 2020 Jul 16;11(7):691. doi: 10.3390/mi11070691 (PMC7408473; doi:10.3390/mi11070691)
Supplement: Supplementary file 1 [file micromachines-11-00691-s001.pdf]

Supplementary Materials: A Microfluidic Probe Integrated Device for Spatiotemporal 3D Chemical Stimulation in Cells

Kenta Shinha, Wataru Nihei and Hiroshi Kimura

Table S1. Detailed conditions for mesh size.

Proximity Min Size	0.1 μm
Max Face Size	10.0 μm
Max Tet Size	10.0 μm
Growth rate	1.2
Elements	9306415

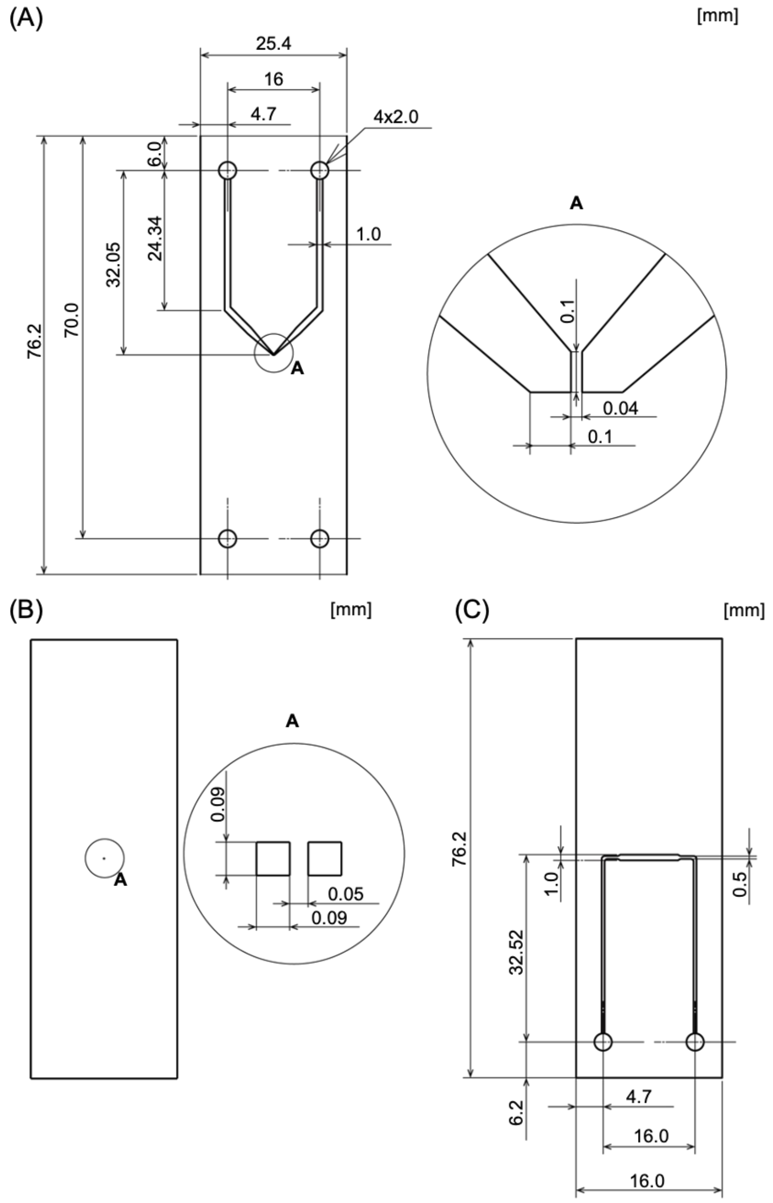

Figure S1. The dimensions of the MFP integrated device designed with CAD software. (A) The upper layer, (B) the middle layer, and (C) the lower layer.

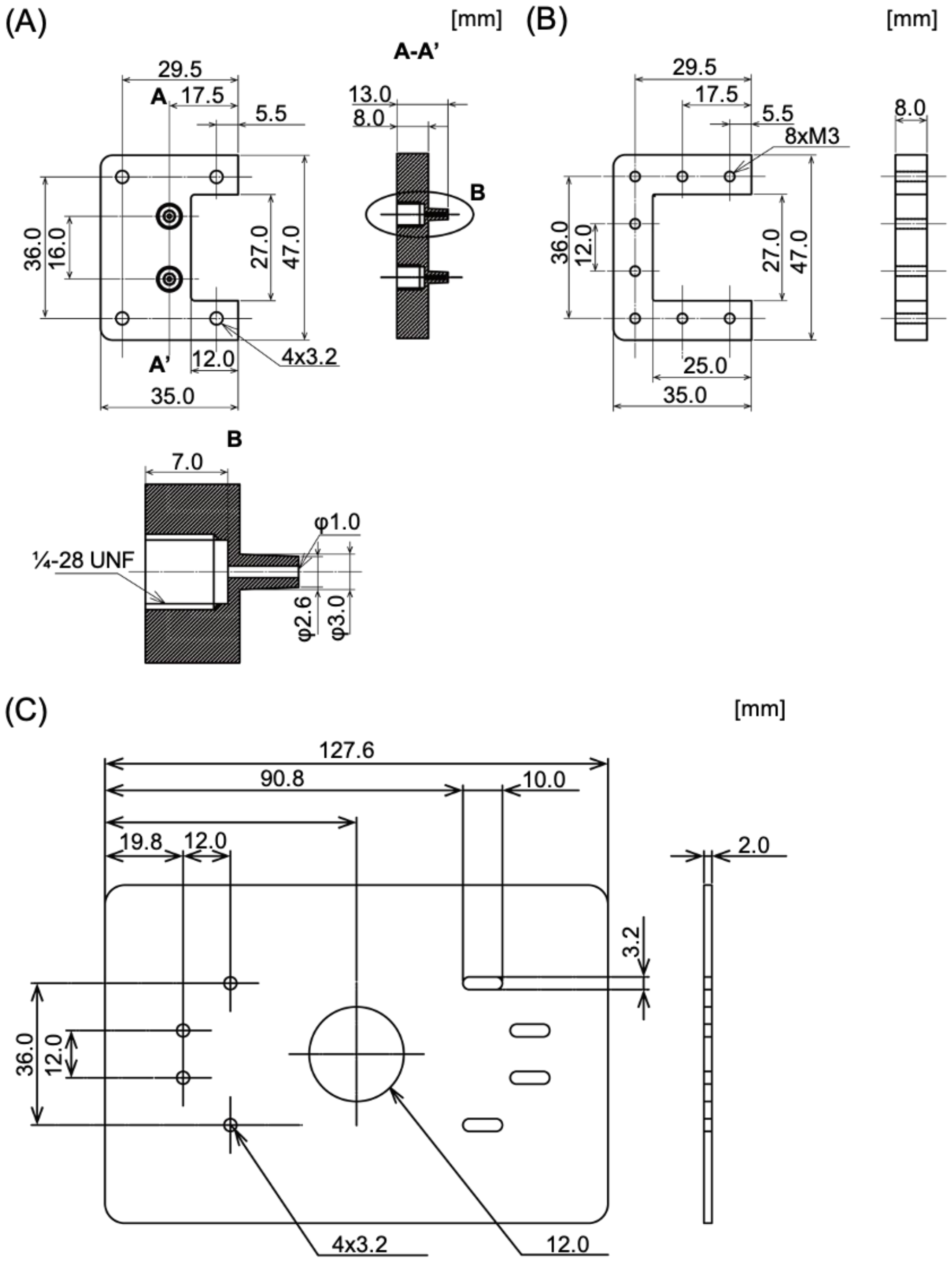

Figure S2. The dimensions of the jig designed with CAD software. (A) The jig that was made of poly ether ether ketone (PEEK). (B) The jig spacer that was made of duralumin. (C) The jig plate that was made of duralumin.

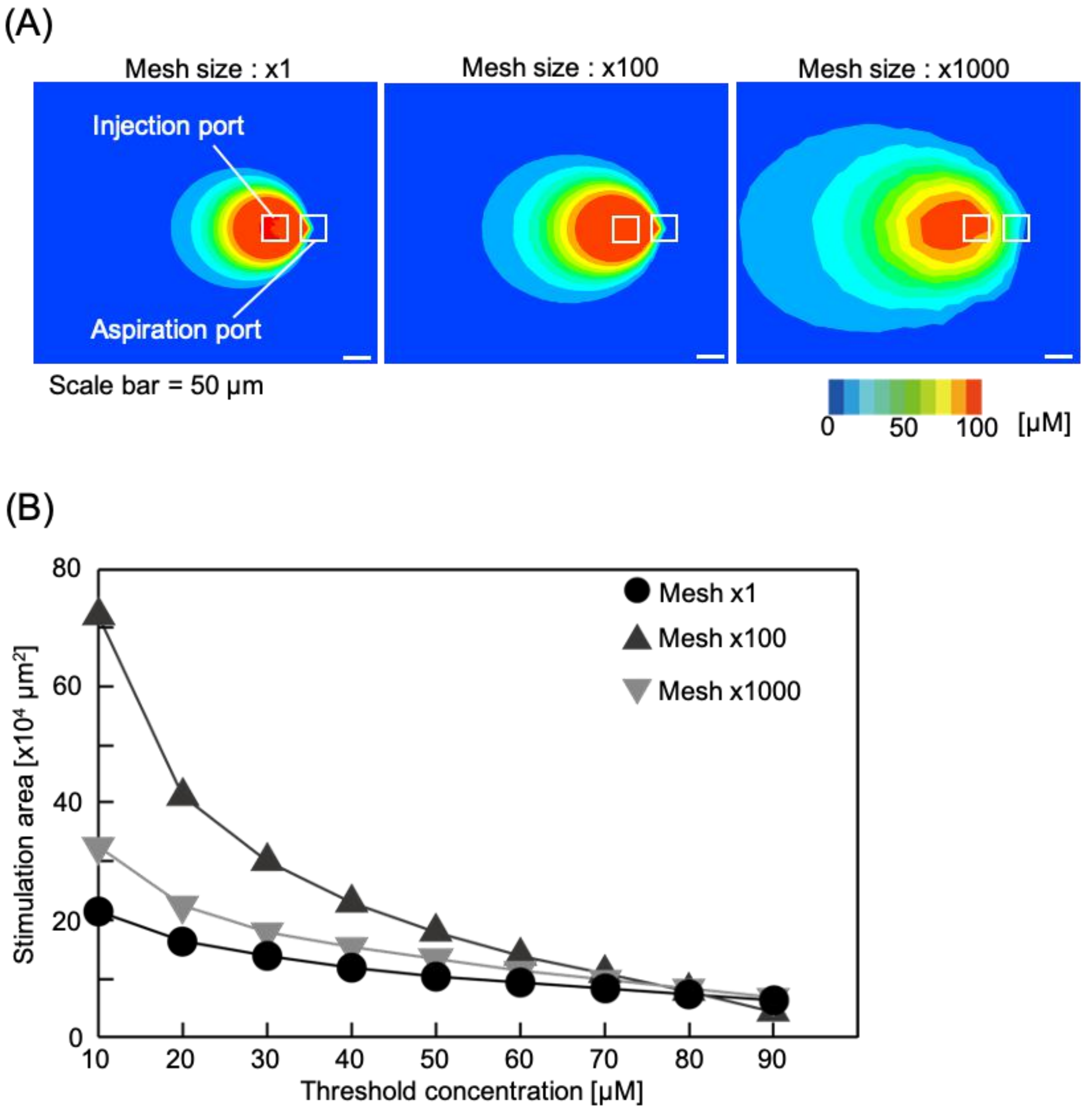

Figure S3. Evaluation of the effect of mesh size on simulation results. (A) Simulation results for the chemical stimulation area when the mesh size was set to 100 times and 1000 times the original size. The flow ratios (Q_I:Q_A) were fixed to 0.33:1.00 μL/min. The chemical stimulation area increases with the mesh size. (B) Graph comparing the stimulation area at the different mesh sizes. The larger the mesh size, the larger is the estimated chemical stimulation area.

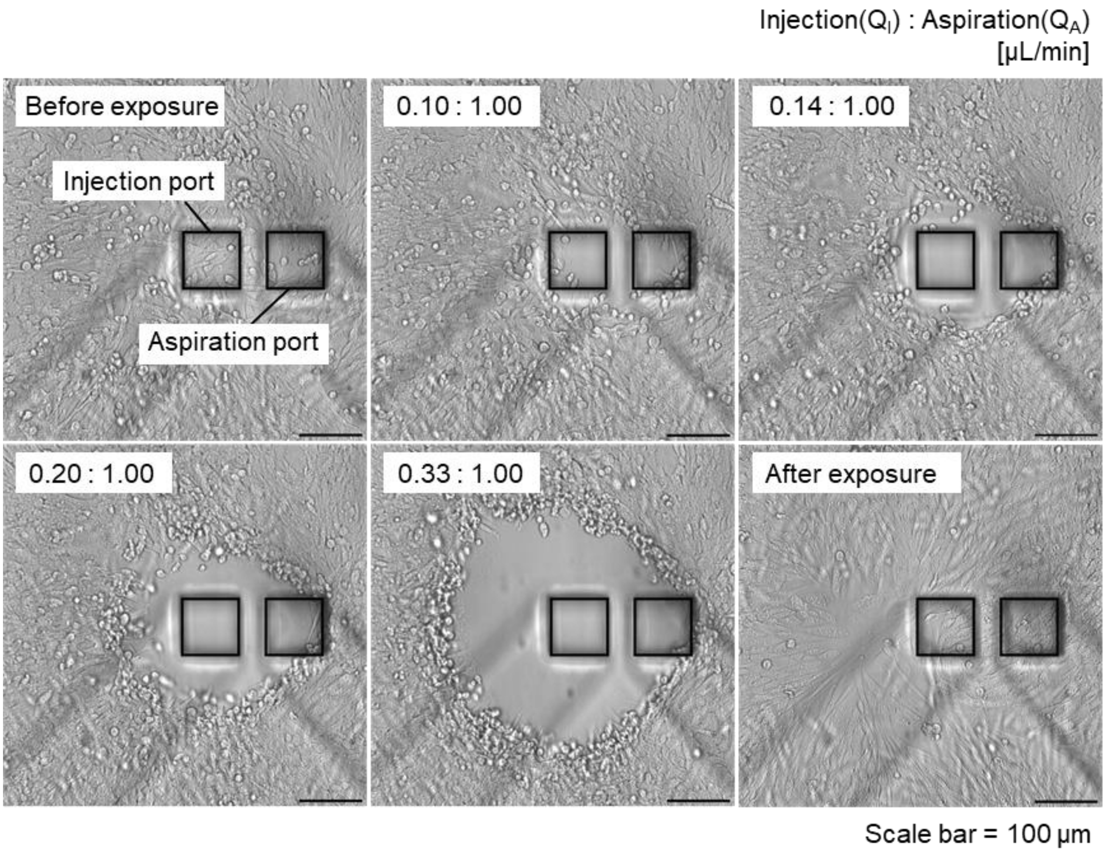

Figure S4. Bright-field images of CHO cells when exposed to trypsin using the MFP on the device to harvest cells. The flow ratios (Q_I:Q_A) were changed gradually from 0.10:1.00–0.33:1.00 µL/min in this experiment. The cells present in the chemical stimulation area lost their adhesion ability, became detached from the substrate, and were collected through the aspiration port of the MFP. The cells surrounding the chemical stimulation area continued to grow and were unaffected by the trypsin treatment. The scale bars are 100 µm.
